# How well are Indonesia’s urban poor being provided access to quality reproductive health services?

**DOI:** 10.1371/journal.pone.0265843

**Published:** 2022-04-12

**Authors:** Elvira Liyanto, Dewi Nuryana, Restu Adya Cahyani, Budi Utomo, Robert Magnani

**Affiliations:** 1 United Nations Population Fund, Indonesia Country Office, Central Jakarta, Indonesia; 2 Knowledge Hub for Reproductive Health Indonesia, Faculty of Public Health, Universitas Indonesia, Depok, West Java, Indonesia; University of Botswana, BOTSWANA

## Abstract

Accommodating the needs of Indonesia’s rapidly growing urban population is essential to reaching national reproductive health goals and international commitments. As in other rapidly urbanizing low- and middle-income countries, satisfying the needs of Indonesia’s urban poor is both a high priority and a significant challenge. In this study, we assessed both how being from urban poor or near-poor households affects the quantity and quality of family planning and maternal health services received and the extent to which differentials had narrowed during the 2012–2017 period. This time interval is significant due to the introduction of a national social health insurance scheme in 2014, establishing the foundation for universal health care in the country. Data from the 2012 and 2017 Indonesian Demographic and Health Surveys were analyzed using logistic and multinomial logit regression. Poverty status was measured in terms of urban household wealth quintiles. For family planning, although urban poor and near-poor women made different method choices than non-poor women, no substantial 2017 differences in contraceptive prevalence, unmet need for family planning or informed choice were observed. However, urban poor women and to a lesser extent near-poor women systematically lagged non-poor urban women in both the quantity and quality of maternal health services received in connection with recent pregnancies. Significant maternal health service gains were observed for all urban women during the study reference period, with gains for poor and near poor urban women exceeding those for non-poor on several indicators. While the deployment of pro-poor interventions such as the national social health insurance scheme is likely to have contributed to these results, evidence suggesting that the scheme may not be influencing consumer health-seeking behaviors as had been anticipated along with continued limitations in public health sector supply-side readiness resulting in service quality issues suggest that more will have to be done.

## Introduction

The United Nations projects that the world will be 66% urban by 2050 with an additional 2.5 billion more people living in cities, 90% of whom will be concentrated in cities in Asia and Africa [1]. Urbanization along with rapid economic growth and persistent or widening inequality are the dominant demographic and social trends in Asia. By 2030, it is projected that the proportion of the population living in urban areas will reach 56% in South-East Asia and 72% in East Asia [2]. This presents a major challenge for governments, who are struggling to adapt vital public services and social protection systems to meet rapidly growing urban demand. The modern health sector, which to large extent has been based upon a rural model of primary health care (PHC), is endeavouring to adapt governance, financing and service delivery arrangements to meet the needs of rapidly expanding urban populations.

The literature is replete with data and studies that document dire circumstances for the urban poor. For example, a meta-analysis of Demographic Health Surveys (DHS) from 31 developing nations indicated that the odds of having a skilled attendant at delivery was 94% lower for women in poorest wealth quintile and five times higher among women with complete primary education [3]. Other studies have established that people living in urban slums experience social, economic, and political exclusion [4–12], have weak social networks [13, 14] which in many cases bars them from society’s basic resources, and have limited access to health insurance [15, 16]. People living in urban slums face much greater challenge to improve their health than other parts of countries despite living in relatively close proximity to health services [5].

The plight of the urban poor has not escaped the attention of the international development community (along with, of course, the governments of rapidly urbanizing poor- and middle-income countries). Several international development initiatives have mobilized funding to better address the family planning and reproductive health needs of the urban poor, including the Bill and Melinda Gates Foundation’s Urban Reproductive Health Initiative (URHI) and follow-on Challenge Initiative, as well as the global FP2020 initiative. Largely positive results have been reported for the URHI initiative in methodologically strong evaluations [17–19]. A recent study in Bangladesh also reported positive results [20], although earlier studies had produced less compelling results [21–25].

Like most East Asian countries Indonesia has experienced rapid urbanization, with the proportion of the population residing in urban areas having increased from 36% in 1995 to 55% in 2019 and is projected to reach 70% by 2045 [26]. Understanding how urbanization is affecting access to and use of reproductive health services are important public policy issues in the Indonesian context, especially given persistently high maternal mortality estimated at 305 maternal deaths per 100,000 live births in 2015 [27]. Reducing maternal mortality and the prevalence of childhood stunting are among the highest priority health goals in the Government of Indonesia’s (GoI) 2020–2024 Medium-Term Development Plan [28].

The key to reaching Indonesia’s national and global targets and aspirations with regard to reproductive and maternal-child health would seem to increasingly lie in accommodating the needs of its growing urban population, most notably the urban poor. To date, however, there have been few systematic analyses undertaken of the magnitude of differential access to and use of reproductive health services in this important population sub-group nor of trends over time. Two of the available studies examined the poor nationally [29, 30], while a third examined urban-rural differentials [31]. The only study focusing on the urban poor specifically was a study of the determinants of hospital childbirth in urban poor communities [32].

In the present study, we assessed how being from a poor or near-poor household in urban areas in Indonesia affected family planning and maternal health service use during the 2012–2017 period and the extent to which GoI efforts in mitigate differentials in service access and use had been successful. In the way of context, the World Bank [33] estimates that between 1993 and 2019, the share of Indonesians living below the national poverty line declined by approximately 60%, reaching 10.6% in 2017 and 9.4% in 2019. Declining poverty levels were characteristic of both urban and rural areas–from 13.5% in 2006 to 7.8% in 2016 in urban areas and from 21.8% to 14.1% in rural areas [33]. However, the pace of poverty reduction after 2010 was only about one-half as rapid (0.3 percentage points per year) as between 2003 and 2010 (0.6 percentage points per year). Even at reduced levels of poverty, however, economic vulnerability remains high–in 2018, 73.9 million individuals (30% of the population) were deemed to either be poor or vulnerable to falling back into poverty [34].

The reference period for the study is significant because of the establishment of two interventions designed to expand access to reproductive health services among the poor. The first, the *Jaminan Persalina* (Jampersal), was initiated in 2011 to provide poor women that did not have health insurance access to and potentially lifesaving maternal health services. The program covered delivery, post-partum family planning, and low-birth weight childcare services, as well referral transport and maternal waiting home costs. The program was suspended in 2014 following the introduction of a national social health insurance scheme and then revived in 2016 to meet the needs of women who “fell through the cracks” in the national social health insurance scheme. The second intervention was the national social health insurance scheme, the *Jaminan Kesehatan Nasional* (JKN), introduced in 2014. JKN coverage had grown to around 70% by 2017 and 82.7% in 2019 [35], with the ultimate goal of achieving universal coverage (originally targeted for 2019). JKN is intended to provide health insurance coverage as well as a social safety net in the form of disability and retirement benefits. Insurance premiums for poor households are paid by the GoI, but coverage is uncertain for the near poor, many of whom work in the informal sector of the economy and thus do not have insurance premiums paid by the government or co-financed by employers. Such workers are the main barrier to achieving universal coverage.

## Materials and methods

All data used in the study derive from the 2012 and 2017 Indonesian Demographic and Health Surveys (IDHS) [36, 37]. A total of 43,852 women of reproductive age were successfully interviewed in the 2012 IDHS and 47,963 in the 2017 IDHS. The IDHS provides detailed data on the socioeconomic and demographic background characteristics of survey respondents as well as on their current and recent contraceptive behaviors and their experiences with pregnancies occurring in the five (5) years prior to the surveys. We focused our analyses on the last pregnancy reported by respondents. Data were available for 35,479 married women of reproductive age, 22,623 current contraceptive users and 15,021 women with recent births in the 2017 IDHS. Corresponding 2012 IDHS figures were 33,291, 20,639, and 14,782, respectively.

The analyses were conducted in two stages. First, we examined differentials in family planning and maternal-child health related behaviors and service utilization for the urban poor, near poor and non-poor operationally defined as the lowest 20% of households with regard to household wealth, the next lowest 20%, and the remaining 60%. The standard Demographic and Health Surveys household wealth index [38] calculated for urban areas only was used to construct urban wealth quintiles. As World Bank analyses [34] indicate that a substantial proportion of Indonesian households that may be classified as non-poor at any given point in time are vulnerable to sliding back into poverty, we opted to focus on the circumstances of the bottom two wealth quintiles that are referred to as poor and near-poor.

The variables considered in the analyses were chosen to assess both the quantity of service use and the quality of services received. The operational definitions of the behaviors, service use measures and outcomes considered in the analyses are provided in Table 1. Differentials by poverty group status (poor, near-poor, non-poor) were assessed via logistic and multinomial logit regression depending upon the measurement scale of the respective variables. The estimated log-odds from the multinomial logit results were converted to odds-ratios to simplify interpretation. Comparisons were made in terms of adjusted odds-ratios. Control variables were added sequentially in two blocks of variables. The first block of control variables consisted of age, education and number of surviving children for family planning outcomes and parity for MCH outcomes. We then added a control variable for health insurance coverage in order to be able to isolate its effects net of the first block of control variables.

**Table 1:** Operational definitions of variables used in the analyses.

Variable	Definition
Age	Respondent’s current age in years
Education	Coded 0 if highest education was none/primary; 1 if junior high school; 2 if senior high school; 3 if academy/university
Number of Living Children	Total number of living children
Parity	Total number of children ever born
Insurance coverage	Coded 0 if no insurance; 1 if national insurance (JKN); 2 if other insurance
Wealth Quintiles	Wealth index. Coded 1 if poorest; 2 if poorer; 3 if middle; 4 if richer; 5 if richest
Method knowledge	Twelve reference contraceptive methods. Coded 0 if total methods known was above the mean (7+); 1 if below the mean
Method information index	Coded 0 if at time of acceptance of current method respondents were told (1) about other family planning methods, (2) about possible side effects and (3) what to do about side effects; Coded 1 if not all information provided
Contraceptive use	Coded 0 if respondent is using modern contraceptive method; 1 if respondent is using traditional method; 2 if no method
Modern method used	Coded 0 if using long term contraceptive method (implant, IUD); 1 if short term method (injection, pill, condom); 2 if permanent method (female and male sterilization)
Unmet need for FP	Unmet need for FP spacing is defined as fecund women who are not using a contraceptive method and (1) want no further children or to wait for two or more years for their next birth, (2) are unsure whether they want another child, (3) want another child but are unsure when to have the birth, (4) are pregnant but wanted current pregnancy later, or (5) are postpartum amenorrhoeic and wanted last birth later. Coded 1 if unmet need; 0 otherwise
Unmet need for modern methods	Coded 1 if unmet need for modern contraceptive methods; 0 otherwise
Number of ANC visits	Coded 0 if above the mean (more than 8 times); 1 if below the mean (1–8 time(s))
Person providing ANC services	Coded 0 if ANC check was performed by a doctor; 1 if a nurse; 2 if a midwife; 3 if traditional birth attendant
Months of pregnancy at first ANC visit	Month of respondents’ pregnancy when ANC at the first time. Coded 0 if below the mean (0–2 month(s)); Coded 1 if above the mean
Number of days iron tablets or syrup taken	Coded 0 if above the mean (more than 106 days); 1 if below the mean (1–106 days)
Person who helped delivery	Coded 0 if doctor; 1 if nurse; 2 if midwife; 3 if traditional birth attendants (TBA); 4 if relative/friend
Place of delivery	Coded 0 if delivery at a health facility; 1 if at home
Caesarean delivery	Coded 0 if respondents giving birth by caesarean section; 1 otherwise
Timing of first check after delivery	Coded 0 if check-up after delivery was done within12 hours; 1 if more than 12 hours; 2 if none
Timing of mother postnatal check	Coded 0 if done within a week; 1 if more than one week; 2 if none
Baby postnatal check within 2 months	Coded 0 if respondent’s baby received a postnatal check-up within 2 months; 1 otherwise

The second stage of the analyses entailed assessing whether urban poor vs. non-poor gaps in reproductive health services use and quality had contracted during the 2012–2017 period. We estimated a series of difference-in-differences (DID) models that contained interactions between poverty group status and survey round. The functional form of the models was as follows:

$$Y_{it}=\alpha_{0}+\beta 1SR_{t}+\beta 2P1_{it}+\beta 3P2_{it}+\beta 4(P1_{it}*SR_{t})+\beta 5(P2_{it}*SR_{t})+\beta_{n}C_{it}+e_{it}$$

Where:

Y_it_ = the outcome of interest for individual i at time t,

SR_t_ = IDHS survey round coded 1 if the observation corresponds to the 2017 IDHS and 0 if the 2012 IDHS,

P1_it_ takes the value of 1 if individual i resided in a household that was in the lowest 20% wealth quintile and 0 otherwise,

P2_it_ takes the value of 1 if individual i resided in a household that was in the 20–40% wealth quintile and 0 otherwise,

(P1_it_*SR_t_) and (P2_it_*SR_t_) are interaction terms between survey round and membership in the respective household wealth quintile groups of interest,

C_it_ is a vector of control variables (see above for a list of the control variables included in the analyses), and

ε_it_ is the error term that represents unobserved (and presumably random) sources of variation.

The main parameters of interest are the coefficients of the interaction terms β4 and β5, which measure the differential survey or time effect for women in the lowest and next lowest wealth quintiles, respectively, in comparison with changes among women classified as being non-poor, the reference category in the regressions, measured by the coefficient β1. The regressions were estimated using modified linear probability models (MLPM) with binary outcomes [39–41]. MLPM was chosen to simplify interpretation. The modifications made to the basic LPM model entailed (1) the calculation of robust standard errors to address heteroscedasticity and (2) the restriction of the variables considered in the analyses to those with probabilities between 10% and 90% to avoid non-linearity issues [42, 43]. Because of the large sample size for the study, non-normality is not a major problem [44, 45]. A comparable MLPM approach was used in a recent study of inequality in reproductive health service utilization and child nutrition outcomes in urban Bangladesh [20]. All analyses were undertaken using STATA Version 16.

## Results

The following brief summary of the structure of the Indonesian health system is provided as context for reader’s considering the study results. The Indonesian health system is a complex, decentralized system in which the central level Ministry of Health (MoH) has authority over health policy, regulations, strategic planning, coordination with other government ministries and the private sector, and monitoring and evaluation. Actual delivery of public sector health services is the responsibility of city and district governments, specifically City/District Health Offices. Public health services are financed via a combination of local revenue, fiscal transfer streams from the central level (some earmarked for particular purposes, some not; some tied to performance targets, but most not), and revenue from the national social health insurance scheme (JKN). Both supply- and demand-side financing schemes are used. Primary care is financed by JKN via capitation (a fixed budget) while hospitals are reimbursed based on case-based groups (CBGs) with no cap on spending (an open-ended budget). The private sector plays a significant role in the delivery of health services in Indonesia. The most recent Health Facility Research Survey (RIFASKES) undertaken in 2019 [46] indicated that there were five times as many private primary health care providers (PHC) facilities (i.e., private clinics and individual physician practices) (53,011) as public PHC providers (10,203). If the number of private midwives also taken into account, there may be up to ten times as many private PHC providers (95,299) as public PHC providers (10,203). The 2019 RIFASKES indicate that only 51% of 13,232 private clinics sampled in the survey were registered with the BPJS-Kesehatan, the organization charged with administering the JKN program, only 13.5% of 39,779 general practitioners, and only 5.9% of 42,288 private midwives. The “JKN “empanelment rate” in 2017, the reference end date for the present study, was even lower.

Table 2 presents full-sample estimates of the variables considered in the study from the 2017 and 2012 IDHS. As may observed, as of 2017 Indonesia fared reasonably well nationally regarding family planning. The contraceptive prevalence rate (CPR) in urban areas was 63.0% and the modern contraceptive prevalence rate (mCPR) was 54.9%. These relatively high figures mask the fact that contraceptive use rates have been stagnant since the early 2000s, and levels of unmet need for both family planning and for modern contraception remain moderately high– 11.3% for use of any method and 19.3% for use of modern contraceptive methods. Awareness of contraceptive methods among survey respondents was high, but the level of informed choice at the time of contraceptive method adoption was quite low by international standards–the Method Information Index (a measure of informed choice) in 2017 of only 30.2 on a scale of 0–100%. Minor improvements between 2012 and 2017 were observed on several indicators, including contraceptive prevalence, the method information index, unmet need for family planning and use of longer acting and permanent contraceptive methods. Due to the large size of the samples considered in the study, even modest change emerged as significant. Accordingly, the significance of observed changes should be assessed on substantive grounds.

**Table 2:** Distributions of variables used in the analysis, married urban women, 2017 and 2012 IDHS^a^.

Variable	2017	2012	p-value
**Methods Known**			
Mean	8.0	7.7	0.000
Median	8.0	8.0	
**Method Information Index**			
3 (Yes to all)	30.2	27.7	0.001
0, 1, 2	69.8	72.3	
**Contraceptive Use**			
Modern	54.9	56.9	0.000
Traditional	8.1	5.2	
None	37.0	37.9	
**Modern Method Used**			
STM	73.7	79.7	0.000
LARC	17.2	13.0	
Permanent	9.1	7.3	
**Unmet need for FP**	11.3	11.8	0.08
**Unmet need for modern methods**	19.3	17.1	0.000
**Number of ANC Visits**			
>8	61.9	57.0	0.000
1–8	38.1	43.0	
**Person who checked ANC**			
Doctors or OB-GYNs	18.6	17.9	0.158
Nurses	0.7	0.5	
Midwives	79.1	80.1	
TBA	1.6	1.5	
**Month of pregnancy first ANC visit**			
Mean	2.0	2.0	0.004
Median	2.0	2.0	
**Number of days iron tablets or syrup taken**			
Mean	114.1	100.6	0.000
Median	90.0	63.0	
**Person who helped delivery**			
Doctor	12.6	15.0	0.000
Nurse	10.1	6.3	
Midwives	62.1	60.4	
TBA	5.3	8.9	
Relative/friends	9.9	9.4	
**Place of delivery**			
Health facility	88.4	78.2	0.000
Home	11.6	21.8	
**Caesarean delivery**			
Yes	23.1	18.1	0.000
No	76.9	81.9	
**Timing of first check after delivery**			
Within 12 hours	87.8	74.1	0.000
> 12 hours	8.7	19.0	
None	3.5	6.9	
**Timing of mother postnatal check**			
Within a week	42.7	67.6	0.000
> 1 week	26.9	5.9	
None	30.4	26.5	
**Baby postnatal check within 2 months**			
Yes	68.0	68.8	0.329
No	32.0	31.2	

A number of positive changes in maternal and child health-related indicators are also apparent from Table 2. These included increased numbers of ANC service visits, number of days that iron tablets or syrup were consumed, proportions of deliveries at health facilities and attended by doctors (and a corresponding decline in home deliveries), and timing of initial post-delivery checks being closer to the time of delivery. However, the proportion of women receiving a postnatal check-up within one week of delivery fell precipitously and room for further improvement is apparent on other indicators as well– 119.6% of recent births were delivered at home, 30.4% of women delivering a birth received no post-natal care visits, and 32.0% of babies did not receive a check-up within two months of birth.

How did the urban poor fare on family planning services and outcomes vis-à-vis the urban non-poor in 2017? Table 3, which displays the odds-ratios for the respected outcomes adjusted for age, education, and number of surviving children, suggests that women residing in poor and near-poor households fare relatively well vis-à-vis non-poor women. While both poor and near poor women had lower levels of contraceptive knowledge, no sizeable differences are observed in terms of informed choice, contraceptive prevalence, or unmet need for family planning. The poorest women were, however, less likely to have been using traditional or permanent contraceptive methods, while the near-poor were more likely to have been using permanent methods than the non-poor. The inclusion of a control variable for health insurance status in general dampens the magnitude of differentials only slightly.

**Table 3:** Adjusted Odds-Ratios of selected family planning outcomes for urban poor vs. urban non-poor women.

Outcome Variables	Adjusted ORs for					
	Age, Education, Number of Living Children			Insurance Coverage^a^		
	Non-Poor	Lowest 20%	Next 20%	Non-Poor	Lowest 20%	Next 20%
**Method knowledge**						
Below mean (Reference–Above mean)	1.00	2.11***	1.52***	1.00	2.03***	1.45***
**Method information index**						
0–2 (Reference–Yes to all)	1.00	1.01	1.15	1.00	0.99	1.12
**Contraceptive use**						
Traditional method (Reference–Modern method)	1.00	0.81*	0.95	1.00	0.82*	0.97
o method being used	1.00	1.03	0.96	1.00	1.03	0.96
**Modern method use**						
Short term (Reference–LARC)	1.00	0.94	1.14	1.00	0.92	1.11
Permanent	1.00	0.75*	1.27*	1.00	0.79	1.33**
**Unmet need for FP**						
Unmet need (Reference–Met need)	1.00	1.01	0.97	1.00	1.00	0.96
**Unmet need for modern method (total)**						
Unmet need (Reference–Met need)	1.00	0.93	0.95	1.00	0.93	0.96

*p<0.05

**p<0.01

***p<0.001.

^a^ Adjusted for age, education, number of living children and health insurance.

The results of comparable analyses of maternal and child health services and outcomes shown in Table 4 point to a very different reality. These data suggest that the quantity and quality of ANC and birth delivery and to lesser extent post-natal care (PNC) services received by poor and to a lesser extent near-poor women were inferior to those received by non-poor women more or less across the board. When age, education and parity are controlled statistically, women from the poorest 20% of households were more than two times more likely than non-poor women to have had below the full sample average number of ANC visits, nine times more likely to have ANC services provided by a traditional birth attendant (TBA), over twice as likely to have fallen below the full sample mean with regard to number of days of iron supplementation, more than three times more likely to have delivered at home, 3.76 times more likely to have their delivery assisted by a TBA, and nearly three times as likely to have delivery assisted by a relative or friend (vs. a doctor), and 28% less likely to have had an initial post-natal check within one week as compared to non-poor women. Similar disparities are observed for near-poor women although the size of the odds-ratios is generally smaller than for the poorest women. As with the above family planning analysis, the inclusion of a control variable for health insurance status only dampens the differentials.

**Table 4:** Adjusted Odds-Ratios of maternal and child health services outcomes for Urban Poor vs. Non-Poor women.

Outcome Variables	Adjusted ORs for					
	Age, Education, Parity			Insurance Coverage^a^		
	Non-Poor	Lowest 20%	Next 20%	Non-Poor	Lowest 20%	Next 20%
**Number of ANC visits**						
Below mean (Reference–Above mean)	1.00	2.36***	1.49***	1.00	2.32***	1.47***
**Person who checked ANC**						
Nurses (Reference—Doctors)	1.00	3.53**	1.77	1.00	3.23**	1.65
Midwives	1.00	3.23***	3.18***	1.00	2.91***	2.95***
TBA	1.00	9.25***	5.65***	1.00	8.03***	5.08***
**Month of pregnancy when first ANC**						
Above mean (Reference–Below mean)	1.00	2.09***	1.43***	1.00	2.04***	1.41***
**Number of days iron tablets or syrup taken**						
Below mean (Reference–Above mean)	1.00	1.75***	1.26**	1.00	1.74***	1.25**
**Person who helped delivery**						
Nurses (Reference–Doctors)	1.00	1.40	1.28	1.00	1.40	1.28
Midwives	1.00	2.11***	1.73***	1.00	2.07***	1.68***
TBA	1.00	3.76***	1.99***	1.00	3.69***	1.94***
Relative/friend	1.00	3.06***	2.52***	1.00	3.01***	2.46***
**Place of Delivery**						
Home (Reference–Health facilities)	1.00	3.22***	1.95***	1.00	3.19***	1.91***
**Caesarean delivery**						
No (Reference–Yes)	1.00	1.83***	1.62***	1.00	1.79***	1.57***
**Timing of first check after delivery**						
> 12 hours (Reference–Within 12 hours)	1.00	0.91	1.05	1.00	0.92	1.06
None	1.00	1.21	1.08	1.00	1.2	1.06
**Timing of mother postnatal check**						
> 1 week (Reference–Within a week)	1.00	0.72***	0.80**	1.00	0.73***	0.82**
None	1.00	1.15	1.05	1.00	1.15	1.05
**Baby postnatal check within 2 months**						
No (Reference–Yes)	1.00	1.02	0.94	1.00	1.02	0.94

*p<0.05

**p<0.01

***p<0.001.

^a^ Adjusted for age, education, parity, health insurance.

**Fig 1:**
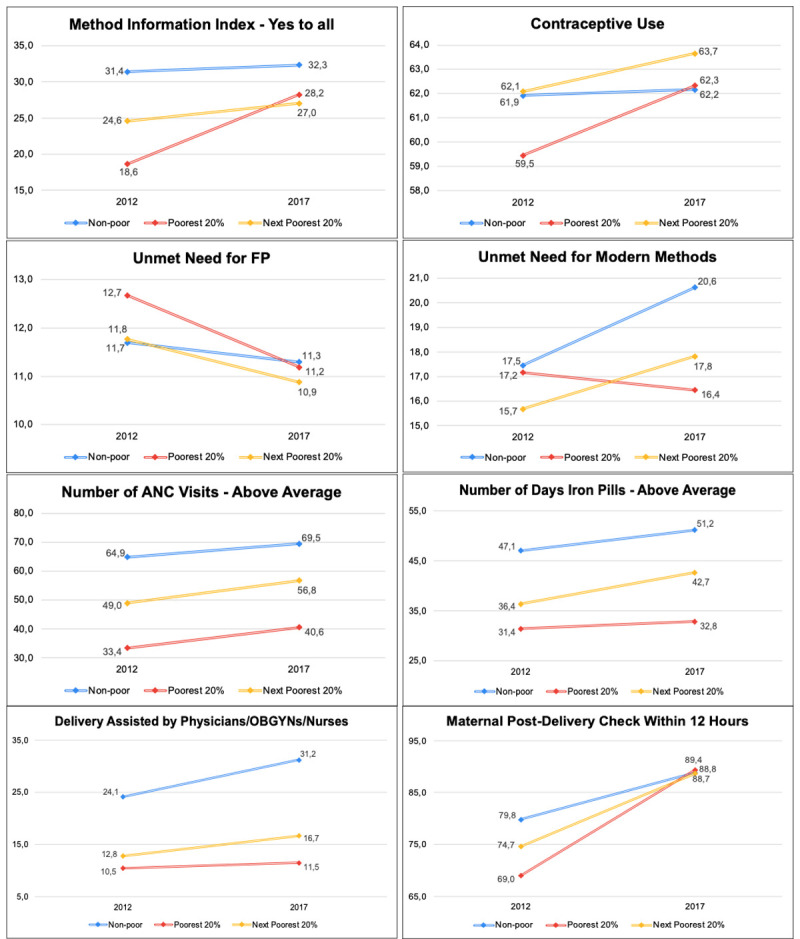
Trends in selected outcomes of family planning and maternal health services, 2012–2017.

Has the plight of poor urban women improved in recent years? Fig 1 and Table 5 provide evidence on this question. Fig 1 compares 2012–2017 trends for non-poor, poor and near-poor women for selected FP and maternal health outcome indicators. Table presents the results of fuller analyses more formal assessment in which we assess the statistical significance of differences in trends for poor and near-poor women vis-à-vis those for non-poor women on the FP and MCH indicators considered and introduce statistical controls for compositional differences in background factors among the three sub-groups of women. The coefficient in the first column of Table 5 indicate the direction and magnitude of changes for non-poor women (labelled as “Time Effect for Non-Poor Women”). Differential “time effects” for poor and near-poor women are shown in coefficients the second and third columns of the table. The coefficients shown in the table were adjusted for differences in age, education and either number of living children or parity depending upon whether a given service outcome was for FP or maternal health, as well as health insurance status.

**Table 5:** Difference-in-differences regression estimates of comparative changes in selected outcomes for non-poor, poor, and near-poor women, 2012–2017.

Outcome Variables	Time Effect for Non-Poor Women		Differential Time Effect for Poorest 20%		Differential Time Effect for Next Poorest 20%	
	Coef	95% CI	Coef	95% CI	Coef	95% CI
Method knowledge–above average^1^	-0.005	-0.023, 0.013	0.045**	0.014, 0.077	0.000	-0.034, 0.034
Method information index–yes to all^1^	-0.007	-0.039, 0.025	0.110***	0.057, 0.162	0.022	-0.033, 0.078
Contraceptive use^1^	0.017	-0.001, 0.035	0.200	-0.016, 0.056	0.014	-0.022, 0.049
Modern method use^1^	-0.013	-0.032, 0.006	0.046*	0.009, 0.084	0.020	-0.017, 0.056
Unmet need for FP^1^	-0.004	-0.016, 0.009	-0.015	-0.040, 0.100	-0.003	-0.026, 0.021
Unmet need for modern methods^1^	0.031**	0.012, 0.051	-0.058**	-0.095, -0.021	-0.016	-0.052, 0.019
Number of ANC visits–above average^2^	0.034**	0.006, 0.062	0.038	-0.016, 0.092	0.025	-0.031, 0.080
ANC services by physician/OBGYN^2^	0.006	-0.017, 0.030	-0.021	-0.050, 0.009	-0.055**	-0.088, -0.023
ANC services by midwives^2^	-0.008	-0.032, 0.016	0.032	-0.001, 0.066	0.048**	0.015, 0.082
Months pregnant at first ANC visit—below average^2^	0.040**	0.016, 0.065	-0.007	-0.061, 0.046	0.028	-0.024, 0.080
Number of days iron pills—above average^2^	0.043*	0.010, 0.076	-0,038	-0.101, 0.025	0.007	-0.057, 0.071
Delivery assisted by physician/OBGYN/nurse^2^	0.058***	0.033, 0.083	-0.061**	-0.099, -0.023	-0.048*	-0.088, -0.007
Delivery assisted by midwives^2^	0.011	-0.018, 0.041	0.063*	0.007, 0.119	0.041	-0.015, 0.098
Post-delivery check within 12 hours^2^	0.083***	0.062, 0.105	0.112***	0.066, 0.158	0.055*	0.009, 0.099
Baby post-natal care check (within 2 months)^2^	-0.046***	-0.072, -0.021	0.054*	0.002, 0.106	0.048	-0.003, 0.099

*p<0.05

**p<0.01

***p<0.001.

^1^Adjusted for age, education, number of living children, and insurance coverage.

^2^Adjusted for age, education, parity, and insurance coverage.

An initial impression from Fig 1 is that the although the 2012–2017 changes were not always large in absolute terms, many of the gains observed in Table 2 were shared to varying degrees by all urban women. Of the eight indicators for which data are shown in the Figure, trends for the poorest women were more favorable than those for non-poor women–informed choice/Method Information Index, contraceptive use, unmet need for modern methods, and timing of post-natal baby check-ups. The sizeable gains in absolute terms with regard to informed choice of contraceptive methods and timing of baby check-ups are especially noteworthy. The relative gain in terms of unmet need for modern contraceptive methods is the result of an increase in unmet need among non-poor women is due to an increase in traditional vs. modern method use among more educated/higher wealth women between the 2012 and 2017 IDHS [36, 37]. Trends were more favorable for non-poor women on two indicators–unmet need for FP and delivery by physicians or nurses although the gains in absolute terms were small. There were no discernible differences in changes between the two rounds of IDHS for the final two indicators–number of ANC visits and consumption of iron pills during pregnancy.

Table 5 displays the results of more refined analyses of comparative changes in family planning and maternal-child health outcomes among non-poor, poor, and near-poor women between the 2012 and 2017 IDHS. The refinements included testing for the statistical significance of differential changes in outcome indicators and the introduction of control variables to control for comparison-group differences in key background factors (age, education, parity, number of living children, and health insurance coverage).

The only statistically significant change in family planning indicators observed for non-poor women was an increase in unmet need for modern contraceptive methods which, as was noted above, was the results on increasing use of traditional methods by more educated, higher household wealth women. IDHS data suggest that this is likely due to concerns over side effects of modern methods [36, 37]. Significant comparative gains are observed for poor women (those in the lowest household wealth quintile) vis-à-vis non-poor women on three indicators. Poor women gained ground regarding contraceptive method knowledge and informed choice at the time of contraceptive method adoption, two results that may well be linked causally. The comparative gain in the third outcome, unmet need for modern methods, is as explained above the result of a shift form modern to traditional methods by non-poor women. No statistically significant differences in changes for near poor vs. non-poor women were observed.

Regarding maternal-child health, statistically significant 2012–2017 changes are observed among non-poor women on six outcomes. Positive trends are observed in terms of numbers of ANC visits, month of pregnancy at first ANC visit, number of days in which iron pills were consumed, delivery by a physician/OBGYN/nurse, and having an initial post-natal check within 12 hours of delivery. A negative change was observed in terms of having an initial post-natal baby check within two months. Statistically significant differential changes are observed for both poor and near poor women. Poor women gained ground on non-poor women with regard to post-delivery maternal checks within 12 hours and initial baby checks within two months, but lost ground with regard to delivery by physicians/OBGYNs/nurses. However, deliveries by TBAs fell from 19% to 11% among poor women during the 2012–2017 period, and by 2017 65% of deliveries were assisted by a midwife, a figure that was not statistically different from that for non-poor women (60%). As with poor women, near poor women lost ground with regard to physician/OBGYN-provided ANC services and physician/OBGYN/nurse assisted deliveries and gained ground with regard to post-delivery maternal checks within 12 hours as compared with non-poor women. The proportion of near poor women receiving ANC services form midwives also increased more rapidly than among non-poor women during the observation period.

## Discussion

Given the rapid rate of urbanization, Indonesia’s ability to reach medium- and long-term family planning and maternal health goals and international commitments is inextricably linked to its ability to serve the needs of the urban poor (and near-poor). The present study sought to assess the magnitude of the challenge ahead and the extent to which progress had been made in recent years.

Our analyses yielded quite different conclusions regarding access to and use of family planning vs. maternal health services. For family planning, the level of contraceptive use and the quality of services received by urban poor and near-poor women did not differ markedly from non-poor women. Both poor and near-poor women did, however, lag behind non-poor women with regard to contraceptive method knowledge and differed in terms of choice of contraceptive method being used. However, urban poor women and to a lesser extent the near-poor systematically lagged behind non-poor urban women in terms of both the quantity and quality of maternal health services received in connection with recent pregnancies.

The observed 2012–2017 trends were, however, encouraging. While FP indicators were stagnant during the reference period for urban non-poor women, the poorest urban women realized statistically significant comparative gains in contraceptive method knowledge, informed choice (an indicator of FP counselling service quality), contraceptive prevalence, and unmet need for modern contraceptive methods.–The findings regarding maternal health and child services were perhaps even more encouraging given the inequities observed in the 2017 IDHS data. Statistically significant 2012–2017 gains were observed for non-poor urban women on six of the nine indicators considered–five in a favorable direction and one negative. Of the five significant positive changes observed for non-poor women, changes for poor women were not significantly different for four of the indicators and the magnitude of gains for poor women exceeded that for non-poor women on one of these (post-delivery maternal checks within 12 hours). A positive gain was also observed for poor women on the one observed significant negative change for non-poor women–post-natal baby checks within two months. The gains for non-poor women were also generally shared by near poor women, who like poor women experienced larger gains on some indicators than non-poor women. The findings are thus consistent with expectations of the pro-poor JKN and Jampersal programs, although the relative gains were for many services modest in magnitude and large differentials in access to and use of most maternal health services remained in 2017.

The study finding that the near poor women, those in the second lowest household wealth quintile, are disadvantaged with regard to the quantity and quality of maternal health services consumed in comparison with non-poor women is an important observation from a policy perspective. The World Bank [47] has suggested that the GoI consider extending payment of JKN insurance premiums to cover informal sector workers, most of whom fall into the bottom two household wealth quintiles. Not only would the implementation of such a policy reduce financial barriers to maternal health service use by near poor households, but it would provide public sector primary health care facilities with a steady stream of additional revenue that could be used to improve the quality of services. The initiative could be largely, if not entirely, financed from savings realized from having smaller JKN operating deficits that have to be covered by the Ministry of Finance.

Why might access to and use of services by urban poor and near poor be different for family planning and maternal health? A key underlying factor is the differential level of success of the national family planning and MCH programs [48]. Although it has been somewhat stagnant since the early 2000s, by 2017 the national contraceptive prevalence rate (CPR) had reached 64% and unmet need for family planning had fallen to 11%, while the modest household wealth quintile differences in both indicators measured in the 2002–03 IDHS [49] had completely disappeared by 2017 [37]. The national MCH program on the other hand has struggled mightily as indicated by a maternal mortality ratio of 305 per 100,000 live births in 2015 [27] that is not commensurate with a country that the World Bank had “graduated” to upper-middle income status prior to a COVID-19 pandemic-related setback. A recent community-level ecological study [50] found that maternal mortality ratios were strongly associated with the proportion of households that were classified as poor but only weak (at best) associations with local health system infrastructure/resource indicators (i.e., numbers of hospitals, community health centers, physicians, nurses, and midwives), a strong indication of program ineffectiveness.

A second contextual issue concerns differences in source of services and payment between family planning and MCH services in Indonesia. Since the early 2000s, the primary source of supply for contraceptive services/supplies for Indonesian women has been the private sector (62–72% range between 2002–03 and 2017), with private midwives being overwhelmingly the most common service/supply source and pharmacies realizing steadily increasing market share (14.1% in 2017). Paying private midwives and pharmacies for contraceptive services/supplies has thus become “normal” for Indonesian women at all levels of household wealth. In contrast, the 2017 IDHS data (and data from earlier rounds of IHDS as well) indicate clearly that poor and near-poor women are unwilling or unable to pay for the same quantity and quality of maternal health services as non-poor women.

There is, however, some indication in the data analyzed for the current study that the introduction of JKM (and perhaps Jampersal) may have begun to close the gap between poor and non-poor urban women in terms of quantity and quality of MCH services received. There are reasons to believe that such gains will continue, perhaps at a faster rate, in the future. First, while the proportion of women in the lowest wealth quintile without health insurance declined from 61% in 2012 to 39% in 2017, nearly 4 in 10 poor women were still without health insurance in 2017, and among those that were insured 19% had private (vs. public) health insurance despite their limited financial means and higher private insurance premium costs. Thus, the increase in the proportion of the poorest women covered by government health insurance between 2012 and 2017 was only 15 percentage points. Second, although government regulations call for all health service providers to participate in the JKN, the “empanelment” of private sector service providers was at an early stage in 2017. This is important as Indonesian women/families appear to have a preference for private sector health services. Data from the 2019 National Socioeconomic Survey (SUSENAS) [51] revealed that among respondents reporting having utilized outpatient services in the month prior to the survey, 60% of outpatient visits occurred at private facilities/health practices. The figures from the 2010 SUSENAS conducted eight years earlier were virtually identical [52]. Other things being equal, the expansion of JKN participation by private sector service providers should offer greater opportunities for poor women to avail of services from preferred private sector providers.

There are, however, several issues that must be addressed if the national social health insurance scheme is to significantly reduce poor-non-poor differentials in use of maternal health services. One issue concerns cumbersome and complex administrative procedures to utilize JKN. The 2019 SUSENAS data provide a sense as to how such administrative procedures may be influencing health seeking behaviors. The survey data showed that among respondents visiting a primary care provider in the month before the survey only one-third paid for the consultation using health insurance cards and two-thirds paid "out-of-pocket" (OOP). The benchmark for assessing these figures is the estimated 70% national JKN coverage in 2017. Among those who paid OOP, 29% had JKN insurance but chose not to use it.

Second, because the primary source of JKN revenue is capitation payments based upon health facility “catchment area” population size, a local residence card is generally necessary to take advantage of JKN enrolment. Thus, migrants and other persons that had yet to establish a legal local residence may be denied covered services despite JKN enrolment. For reasons described above, this has a larger impact on use of maternal health than family planning services.

Third, government insurance eliminates some but not all costs of service use. It does not, for example, cover transportation costs to health facilities or opportunity costs associated with long waits for services and long payment reconciliation times.

Finally, while government health insurance may well increase maternal health service utilization by poor urban women by reducing service costs, it will not necessarily lead to increased access to quality services as the Indonesian public health system is struggling to provide quality services to an ever-growing client base as the country scales up to universal health care (UHC) [53]. Indeed, the 2019 RIFASKES data [46] revealed significant remaining supply-side service readiness gaps for many health programs, including maternal-child health, in particular at the Community Health Center (Puskesmas) level, the entry point into the public health system. An earlier study [48] pointed to the need for improved midwife training and reverse/upward task shifting of at least some services from midwives to doctors and nurses as the keys to reducing maternal mortality in Indonesia. Although some progress may have been made, this almost certainly remains a work in progress.

An important limitation of the study should be noted. Because the datasets analyzed did not permit the identification of individual cites/districts (only provincial and sample cluster IDs were available), we were only able to analyze urban areas in Indonesia in the aggregate. It is entirely possible that large and smaller cities might differ with respect to barriers or facilitating factors to healthcare service access and use. For example, residents of smaller regional cities may have better access to services due to differences in distance to health facilities, ease and cost of travel, stronger social network connections and support, and better coordination among service providers playing different roles in the service delivery process. This is an important area for future research in Indonesia given the economic and socio-cultural diversity of Indonesian cities.

In closing, it is to be noted that a recent “critical scoping review” of urban family planning in LMICs [54] identifies four (4) priority themes or areas for future research that in the view of the authors would increase our understanding of the challenges involved in improving the delivery of family planning (and maternal health) services in different urban contexts. These themes resonate with the issues addressed in and the findings of the present study. They include (1) the need to better understand to role of “neighborhoods” (i.e., locality and place) in magnifying or mitigating the impact of poverty and the heterogeneity of these effects both within and across countries; (2) the need for more in-depth understanding of governance-related issues–more precisely, how should family planning and maternal health services be inserted into or strengthened within the complex array of urban actors and institutions; (3) the need for a deeper understanding of the extent to which migrants are or are not assimilated into urban communities and the implications of this for access to health services, and (4) wider adoption of “resilience” (vs. "vulnerability") frameworks and perspectives in research to better inform the issue of how family planning and maternal health services can form part of strategies to enhance the agency of the urban poor and near poor to be more resilient in the face of short- and long-term risks and shocks.

## Conclusions

The family planning needs of urban poor and near poor women in Indonesia appear to be being relatively well accommodated. This is not the case for maternal health services. However, our analyses suggest that both poor and near poor women experienced comparative gains with regards to maternal health services between 2012 and 2017. Although the GoI anticipates that the deployment of pro-poor interventions such as the national health insurance scheme JKN will help reduce remaining poor-non-poor differentials and make a dent in Indonesia’s unacceptably high level of maternal mortality, evidence suggesting that the JKN may not be influencing consumer health seeking behaviors in the ways anticipated along with continued limitations in public health sector supply-side readiness resulting from a comparatively low level of public spending on health suggest that more will have to be done.
